# Exploring Physical Activity, Sleep, and Nutrition’s Role in Fatigue Among Post-COVID-19 Patients

**DOI:** 10.3390/nu16234056

**Published:** 2024-11-26

**Authors:** Isabell Pink, Miriam Wiestler, Lea Pueschel, Jannik Ruwisch, Nora Drick, Lennart Boblitz, Michele Scharbau, Tobias Welte, Sven Haufe, Uwe Tegtbur, Momme Kück, Arno Kerling, Sebastian Beyer

**Affiliations:** 1 Department of Respiratory Medicine and Infectious Diseases, Hannover Medical School, 30625 Hannover, Germany; ruwisch.jannik@mh-hannover.de (J.R.); drick.nora@mh-hannover.de (N.D.); boblitz.lennart@mh-hannover.de (L.B.); scharbau.michele@mh-hannover.de (M.S.); welte.tobias@mh-hannover.de (T.W.); 2 Department of Internal Medicine, Division of Gastroenterology, Hepatology, Infectious Diseases and Endocrinology, Hannover Medical School, 30625 Hannover, Germany; wiestler.miriam@mh-hannover.de (M.W.); pueschel.lea@mh-hannover.de (L.P.); tegtbur.uwe@mh-hannover.de (U.T.); 3 Department of Rehabilitation and Sports Medicine, Hannover Medical School, 30625 Hannover, Germanykerling.arno@mh-hannover.de (A.K.); beyer.sebastian@mh-hannover.de (S.B.)

**Keywords:** fatigue, post-COVID, nutrients, daily life restrictions

## Abstract

**Background/Objectives:** A significant number of patients experience prolonged symptoms following COVID-19 in particular cases of fatigue. Yet, pathomechanisms of COVID-19-related fatigue remain unclear. **Methods:** This study investigated patients after confirmed SARS-CoV-2 infection (PC) with relevant fatigue according to the Fatigue Assessment Scale (≥22 points) to rule out malnutrition as a driving factor for fatigue and to evaluate daily activity and sleep characteristics. Dietary behavior was recorded through food diaries and physical activity by self-reported (questionnaires) and objective (activity tracker) outcomes. Data were collected over a 7-day period and compared with a healthy control group (HC). A subgroup analysis of patients with fatigue and severe fatigue, as well as a sex-specific analysis, were included. **Results:** No significant differences in dietary intake were observed, but an indication toward a healthier Mediterranean diet in PC patients with a median Mediterranean Diet Score of 4 (IQR 3, 5) in HC vs. 5 (IQR 3, 6) in PC (*p* = 0.24). There were also no differences in physical activity, either by objective or subjective measures. However, the median sleep duration was 49 min longer in PC patients (*p* = 0.003). **Conclusions:** In conclusion, malnutrition did not significantly contribute to fatigue, yet patients with COVID-19-related fatigue showed increased sleep duration. As sleep characteristics play a crucial role in mental and physical wellbeing, the association of sleep, physical activity, and fatigue should be evaluated in further studies.

## 1. Introduction

Fatigue is a common symptom among patients who experience ongoing sequels after coronavirus disease 2019 (COVID-19). These patients are often referred to as having post-acute sequels of COVID-19 (PASC). Up to 50 percent of patients suffer from this life-restricting burden following the diagnosis of COVID-19, the etiology of which remains still unclear [1]. Autoimmune processes, persisting virus-antigens, and other multiple etiologies have been described for fatigue like other viral infections, especially Epstein Bar Virus (EBV) or human immune deficiency virus (HIV), as well as cancer, for example, breast cancer [2,3,4]. Until now, the pathogenesis of this phenomenon remains unclear.

Patients with PASC exhibit a reduced daily exercise capacity and experience post-exertional malaise [5,6]. In regard to supportive therapeutic options for fatigue, it is imperative to ascertain whether other causes of physical or mental capacity impairment are present, particularly an inadequate intake of calories or malnutrition, as well as an insufficient duration of sleep. Fatigue, nutrition, and circadian rhythm have been suggested as possible mutually linked factors. For one, metabolic disturbances in energy metabolism may underlie exercise intolerance in chronic fatigue as a post-COVID symptom. Loss of appetite, which is another symptom, may also contribute to fatigue [7]. Diarrhea and vomiting can also be post-COVID symptoms and thus affect exercise tolerance [8]. It has been suggested that the development of chronic fatigue (CF) in PASC is exacerbated by a pre-covid circadian rhythm disorder and inadequate exposure to light [9]. In an observational study from 2021, 201 post-COVID patients who experienced persistent fatigue received nutritional supplements for 28 days. The study showed significant results for improvement in chronic fatigue and overall quality of life [10]. The impact of nutrition on exercise capacity has already been assessed [11]. Walnik et al. showed an association between a higher dietary fiber intake and enhanced physical performance among German students when compared to the general population in Germany [12]. Sleep disturbance is a component of fatigue and a principal criterion of chronic fatigue syndrome (CFS) [13]. Additionally, sleep disturbance is prevalent in post-COVID patients, which has an impact on quality of life and performance [14,15,16]. A small prospective observational study conducted in Chile revealed that 60% of the 60 participants exhibited symptoms of sleep apnea [17].

At present, no causal therapy for fatigue is available, and the impact of special diets or taking dietary supplements is less well-understood. This study was initiated to evaluate the dietary behavior, exercise, and sleep duration of PASC patients with fatigue (PC) in comparison with a historical healthy control group (HC), with the aim of elucidating the potential role of nutrition and sleep quality in the physical and mental impairments frequently observed in these patients.

## 2. Materials and Methods

This is a secondary, exploratory analysis of a randomized controlled trial (RCT) testing the impact of an online-guided intervention on the mental and physical capability of PACS patients [18] (registration trial number: DRKS00026245).

In brief, patients with a previous infection of severe acute respiratory syndrome coronavirus 2 (SARS-CoV-2) confirmed by polymerase chain reaction (PCR < 30 cycles threshold) were included in the study between September 2021 and June 2022. Furthermore, patients were required to meet the following criteria: age ≥ 18 years, persistence of symptoms following a diagnosis of SARS-CoV-2 infection, and a fatigue score of ≥22 on the Fatigue Assessment Scale (© FAS Fatigue Assessment Scale: ILC care foundation http://www.ildcare.nl (accessed on 12 October 2024).

Patients who met any of the following criteria were excluded from participation: current involvement in another intervention study, presence of another clinically relevant acute or chronic infection, pregnancy, surgery less than eight weeks prior, and any medical conditions or functional impairments that, in the opinion of the examining physician, precluded participation in a physical training intervention. Written informed consent was obtained from participants before study inclusion.

A total of 46 patients were recruited from the outpatient clinics of the Department of Respiratory Medicine and Infectious Diseases and the Department of Rehabilitation and Sports Medicine at Hannover Medical School.

The FAS consists of ten questions, 5 concerning physical and 5 concerning mental exhaustion. Every question is answered with 1–5 points with respect to the frequency of occurrence. A score of 22 or above indicates the presence of fatigue, while a score of 35 or above indicates the presence of extreme fatigue [19,20,21]. Cronbach’s alpha of this questionnaire is 0.87 [22].

The groups were matched for body mass index (BMI), sex, and age. All patients were asked to fill out a 7-day food diary, and their daily activity and sleep behavior were tracked by a wearable device.

Patients were requested to complete the validated Freiburger Activity Questionnaire, which is designed to ascertain the total and exercise-related physical activity of adults, both of which are quantified as metabolic equivalents of task (MET)-hours per week [23,24]. This instrument shows average to very good reliability (r = 0.5–0.9) and a moderate validity (r = 0.3–0.4) [24].

Moreover, the patients were requested to complete the Short Form-36 Health Survey (SF-36) [25]. The SF-36 is a tool for measuring health-related quality of life. The 36 items that comprise the SF-36 are designed to reflect eight domains of health, including physical functioning, physical role, pain, general health, vitality, social functioning, emotional role, and mental health. The range is 0–100, with higher scores indicating superior quality of life. Furthermore, a physical and mental composite score can be calculated. The total mental composite score was used to determine the mental and psychological well-being. The German version of the SF-36 exhibits high discriminant item validity in all samples analyzed, with a success rate of over 90% in scaling. Additionally, Cronbach’s alpha reliabilities are above the criterion of 0.70 in all but one scale.

The food diary was subjected to analysis and review by dietitians for macronutrient and micronutrient content using professional nutrition analysis software (DGE-PC professional Version 5.1.0.048, DGE; Bonn, Germany). The reference values are based on the recommendations of the German Nutrition Society (Deutsche Gesellschaft für Ernährungsmedizin, DGE) [26]. To assess Mediterranean diet adherence in all patients, a Mediterranean diet score (MDS) was calculated following the validated method of Trichopoulou et al. [27]. The MDS ranges from 0 to 9, with 1 point awarded for (a) consumption of positively associated foods above the sex-specific mean and (b) consumption of negatively associated foods below the sex-specific mean. A higher score indicates a higher degree of adherence to the Mediterranean diet.

Both groups were equipped with an activity monitor (Forerunner 35 or Forerunner 45, Garmin, Garching, Germany, or Sensewear, Bodymedia, Germany) and were instructed to wear the monitor for one week. The number of steps taken per day, the amount of active energy expenditure, and the duration of sleep were recorded.

### Statistics

Data were first initially evaluated for normality of distribution using the Shapiro–Wilk test. In order to facilitate a descriptive analysis, the absolute frequencies were calculated for categorical variables. For continuous variables, the median and interquartile range (IQR, 25th to 75th percentile) are provided. Group differences were evaluated using the Kruskal–Wallis test for non-parametric data and Pearson’s Chi-square test for categorical variables. A correlation analysis was conducted to examine the relationship between the statistically significant different parameters with the data of the PC group using the Spearman correlation coefficient. To account for multiple comparisons, the Bonferroni correction was applied.

Moreover, in the subgroup analysis of PC patients, a cut-off value of ≥35 points on the FAS was employed to differentiate between patients with fatigue and those experiencing extreme fatigue. In addition, the FAS result was categorized into physical and mental fatigue. There are no specific threshold values defined for this subdivision, but questions 1, 2, 4, 5, and 10 of the FAS are aimed to reflect physical fatigue, and the remaining 5 questions are about mental fatigue [28].

A two-sided significance level was set to 5%. The statistical analysis was performed with R Studio (Version 2024.09.0-375, Posit Software, Boston, MA, USA).

## 3. Results

Table 1 summarizes the different statistical comparisons between the PC and HG groups.

### 3.1. Comparison Between PC and HC Group

Table 2 presents an overview of the patients’ baseline parameters and self-reported activities, the mental composite score of the SF-36 questionnaire, and the activity monitor data for the PC and HC groups. The macronutrient and micronutrient profiles of both groups, alongside the DGE reference values, are shown in Table 2. Approximately two-thirds of the patients were female (77% and 74%), with a median age of 47 years in both groups (*p* = 0.87) and a median Body Mass Index (BMI) of 26 kg per square meters (kg/m^2^) in the HC group and 25 kg/m^2^ in the PC group (*p* = 0.87). As the groups were matched for sex, age, and BMI, with respect to these variables, no significant differences were observed between the groups. Moreover, no significant discrepancy was observed in the reference values for caloric intake, the overall caloric intake, and the ratio of energy intake to the target values.

#### 3.1.1. Freiburger Activity Questionnaire and SF-36 Mental Composite Score

No significant differences were observed in the self-reported total physical and sports-related activities of the participants between the two groups. However, PC patients reported a significantly lower mental composite score in the SF-36 questionnaire (43 vs. 54; *p* < 0.001).

#### 3.1.2. Activity Monitor Data

The two groups exhibited comparable steps per day and active energy expenditure. However, the sleep duration differed significantly, with PC patients demonstrating a mean sleep duration that was approximately 49 min (IQR 41, 64; *p* = 0.003) longer per night than that of the HC group patients.

**Table 2:** Baseline characteristics, self-reported activity, and wearable data of PC and HC group.

Macro- and Micronutrients (Median (Q1, Q3))	Control Group (N = 46)	Post-COVID (N = 46)	Total (N = 92)	*p* Value	Reference Value (DGE) [14]
**Sex**				0.81 ^1^	
female (n /%)	35 (76%)	34 (74%)	69 (75%)		
male (n /%)	11 (24%)	12 (26%)	23 (25%)		
**Age [years]**	47 (40, 53)	47 (39, 53)	47 (40, 53)	0.87 ^2^	
**Body mass index [kg/m²]**	26 (23, 30)	25 (23, 30)	26 (23, 30)	0.87 ^2^	
**Freiburger Activity Questionnaire [MET hours/week]**					
**Sports-related activities**	3.5 (0, 16)	2.0 (0, 7.5)	3.0 (0, 10)	0.47 ^2^	
Missing	2	8	10		
**Total activity**	26 (16, 36)	25 (12, 34)	26 (13, 34)	0.59 ^2^	
Missing	2	10	12		
**SF-36—mental composite score**	54 (51, 57)	41 (29, 50)	50 (37, 55)	**<0.001 ^2^**	
Missing	1	0	1		
**Activity monitor data**					
**Steps per day [steps]**	7400 (6300, 11,400)	6800 (5700, 9500)	7200 (5800, 10,200)	0.10 ^2^	
Missing	15	3	18		
**Measured active energy expenditure [kcal]**	446 (336, 820)	537 (309, 630)	483 (310, 722)	0.43 ^2^	
Missing	15	3	18		
**Measured sleep duration [min]**	437 (389, 476)	486 (453, 517)	466 (421, 508)	**0.003 ^2^**	
Missing	19	3	22		
**Reference value of energy intake [kcal]**	2400 (2100, 2700)	2500 (2300, 2700)	2500 (2200, 2700)	0.91 ^2^	
Missing	1	0	1		
**Energy intake [kcal]**	1800 (1400, 2100)	1700 (1400, 2100)	1700 (1400, 2100)	0.91 ^2^	10–20
Missing	1	0	1		
**Energy intake / Reference value [Ratio]**	0.72 (0.56, 0.90)	0.69 (0.56, 0.80)	0.70 (0.56, 0.82)	0.68 ^2^	
Missing	1	0	1		
**Fat [%]**	34 (30, 37)	37 (33, 41)	35 (31, 39)	**0.02 ^2^**	30
Missing	1	0	1		
**Fat [g]**	64 (51, 75)	67 (59, 88)	66 (53, 86)	0.18 ^2^	
Missing	1	0	1		
**Cholesterol [g]**	0.3 (0.2, 0.4)	0.3 (0.2, 0.4)	0.3 (0.2, 0.4)	0.40 ^2^	
Missing	1	0	1		
Saturated fatty acids [%]	14 (12, 15)	13 (11, 16)	14 (11, 16)	0.45 ^2^	
Missing	1	0	1		
**Saturated fatty acids [g]**	26 (20, 32)	27 (21, 35)	26 (20, 34)	0.58 ^2^	
Missing	1	0	1		
**N-3 Fatty acids (omega-3) [%]**	0.7 (0.6, 0.8)	0.8 (0.6, 1.1)	0.7 (0.6, 1.0)	**0.01 ^2^**	0.5
Missing	1	0	1		
**N-6 Fatty acids (omega-6) [%]**	4.5 (3.8, 5.2)	5.2 (4.2, 6.2)	4.6 (4.0, 5.7)	**0.008 ^2^**	2.5
**Mediterranean Diet Score [points]**	4 (3, 5)	5 (3, 6)	4 (3, 6)	0.24 ^2^	
Missing	20	5	25		
**Carbohydrates [%]**	46 (41, 49)	45 (40, 48)	45 (40, 48)	0.28 ^2^	>50
Missing	1	0	1		
**Carbohydrates [g]**	180 (150, 240)	180 (160, 240)	180 (150, 240)	0.96 ^2^	
Missing	1	0	1		
**Protein [%]**	18 (16, 19)	17 (15, 20)	18 (15, 19)	0.31 ^2^	
Missing	1	0	1		
**Protein [g]**	72 (60, 89)	71 (63, 88)	72 (60, 89)	0.96 ^2^	47–55
Missing	1	0	1		
**Fiber [g]**	20 (17, 24)	22 (19, 29)	21 (18, 28)	0.07 ^2^	≥30
Missing	1	0	1		
**Sucrose [%]**	10 (7, 13)	10 (8, 12)	10 (8, 12)	0.97 ^2^	<10
Missing	1	0	1		
**Sucrose [g]**	40 (28, 57)	43 (33, 53)	42 (31, 54)	0.73 ^2^	61–78
Missing	1	0	1		
**Alcohol [g]**	3.3 (0.4, 8.3)	1.6 (0.03, 5.2)	2.5 (0.12, 6.7)	**0.01 ^2^**	20
Missing	1	0	1		
**Water [liter]**	2.8 (2.2, 3.3)	3.0 (1.9, 3.4)	2.8 (2.1, 3.3)	0.96 ^2^	
Missing	1	0	1		
**Phosphorus [mg]**	1100 (1.5, 1300)	1200 (1000, 1400)	1200 (950, 1400)	**0.03 ^2^**	550
Missing	1	0	1		
**Iron [mg]**	12 (10, 14)	12 (11, 14)	12 (10, 14)	0.55 ^2^	11–16
Missing	1	0	1		
**Zinc [mg]**	11 (8, 12)	10 (9, 12)	10 (8, 12)	0.97 ^2^	7–16
Missing	1	0	1		
**Copper [mg]**	1.8 (1.6, 2.1)	1.8 (1.5, 2.4)	1.8 (1.5, 2.3)	0.80 ^2^	1.0–1.5
Missing	1	0	1		
**Manganese [mg]**	4.3 (3.6, 5.6)	4.2 (3.5, 6.5)	4.2 (3.6, 5.7)	0.68 ^2^	2.0–2.5
Missing	1	0	1		
**Fluoride [mg]**	0.9 (0.7, 1.1)	0.9 (0.7, 1.2)	0.9 (0.7, 1.1)	0.64 ^2^	2.7–3.5
Missing	1	0	1		
**Iodine [µg]**	110 (0.2, 140)	88 (69, 160)	100 (67, 150)	0.29 ^2^	200
Missing	1	0	1		
**Vitamins—fat-soluble**					
**Retinol equivalent [mg]**	1.0 (0.7, 1.3)	1.0 (0.7, 1.4)	1.0 (0.7, 1.3)	0.56 ^2^	0.70–0.85
Missing	1	0	1		
**Vitamin A (Retinol) [mg]**	0.3 (0.2, 0.4)	0.3 (0.2, 0.4)	0.3 (0.2, 0.4)	0.89 ^2^	
Missing	1	0	1		
**Vitamin D (Calciferol) [µg]**	2.5 (1.6, 5.8)	3.7 (1.6, 5.7)	2.7 (1.6, 5.8)	0.41 ^2^	20
Missing	1	0	1		
**Vitamin E (Tocopherol) [mg]**	11 (9, 14)	12 (10, 16)	12 (9, 15)	0.13 ^2^	12–14
Missing	1	0	1		
**Vitamin K [µg]**	220 (160, 310)	260 (190, 340)	250 (160, 330)	0.26 ^2^	60–70
Missing	1	0	1		
**Vitamins—soluble**					
**Vitamin B1 (Thiamin) [mg]**	1.2 (1.0, 1.6)	1.3 (1.0, 1.7)	1.3 (1.1, 1.6)	0.56 ^2^	1.0–1.2
Missing	1	0	1		
**Vitamin B2 (Riboflavin) [mg]**	1.4 (1.3, 1.7)	1.4 (1.2, 1.6)	1.4 (1.2, 1.7)	0.46 ^2^	1.1–1.4
Missing	1	0	1		
**Niacin equivalent [mg]**	28 (25, 33)	28 (24, 36)	28 (24, 36)	0.86 ^2^	12–15
Missing	1	0	1		
**Pantothenic acid [mg]**	4.3 (3.7, 5.3)	4.4 (3.9, 5.6)	4.3 (3.7, 5.4)	0.52 ^2^	
Missing	1	0	1		
**Vitamin B6 (Pyridoxin) [mg]**	1.5 (1.3, 1.7)	1.6 (1.3, 1.9)	1.5 (1.3, 1.9)	0.32 ^2^	1.4–1.6
Missing	1	0	1		
**Biotin [µg]**	44 (35, 55)	49 (38, 58)	45 (37, 56)	0.25 ^2^	40
Missing	1	0	1		
**Folate [µg]**	190 (0.3, 240)	230 (200, 310)	220 (170, 290)	**0.001 ^2^**	300
Missing	1	0	1		
**Vitamin B12 (Cobalamin) [µg]**	4.5 (3.2, 6.2)	4.7 (3.8, 6.5)	4.5 (3.6, 6.5)	0.73 ^2^	4
Missing	1	0	1		
**Vitamin C (Ascorbic acid) [mg]**	120 (89, 160)	150 (110, 210)	140 (100, 170)	**0.03 ^2^**	95–100
Missing	1	0	1		
**Minerals—Macronutrients**					
**Sodium [g]**	2.7 (2.3, 3.0)	2.5 (1.9, 3.2)	2.6 (2.0, 3.2)	0.36 ^2^	1.5
Missing	1	0	1		
**Potassium [g]**	2.7 (2.4, 3.1)	2.9 (2.4, 3.4)	2.8 (2.4, 3.3)	0.41 ^2^	4
Missing	1	0	1		
**Calcium [g]**	0.9 (0.8, 1.1)	0.9 (0.7, 1.0)	0.9 (0.7, 1.1)	0.50 ^2^	1
Missing	1	0	1		
**Magnesium [g]**	0.3 (0.3, 0.4)	0.3 (0.3, 0.4)	0.3 (0.3, 0.4)	0.58 ^2^	3.0–3.5
Missing	1	0	1		

^1^ Pearson’s Chi-squared test; ^2^ Kruskal-Wallis rank sum test, bold *p*-values highlight significant differences between study groups.

#### 3.1.3. Nutrient Intake

PC patients had a significantly elevated percentage of fat intake, accompanied by a higher ratio of unsaturated fatty acids, including omega-3 and omega-6. The percentage of fat consumed was in excess of the recommendations set forth by the DGE for both groups. Patients in the HC group consumed significantly greater quantities of alcohol than those in the PC group (3.3 g in HC vs. 1.6 g in PC group; *p* = 0.01). However, the amount consumed by both groups was well below the recommendations of the DGE. Further evaluation of adherence to a Mediterranean diet was conducted using the Mediterranean Diet Score, which demonstrated at least numerical trends towards higher adherence to the Mediterranean diet in PC patients. However, this did not reach statistical significance. No significant difference was observed in carbohydrate and protein intake between the two groups despite the protein intake exceeding the DGE reference values in both groups. Both groups demonstrated a high intake of water, with an average of 2.8 and 3.0 L per day (*p* = 0.96), respectively. However, no significant difference was observed between the groups. Further details are shown in Table 2.

#### 3.1.4. Vitamins and Minerals

There was no statistically significant difference in micronutrient intake of vitamins between the two groups, with all details shown in Table 2, although there was a numerical trend towards higher intake of vitamins D and K among the PC patients. With regard to water-soluble vitamins, patients with prostate cancer exhibited a significantly higher intake of folate and vitamin C. However, the folate intake of these patients remains below the recommended daily intake of 300 µg. PC patients exhibited a higher intake of phosphorus; however, both groups demonstrated a daily intake that exceeded the general recommendations of the DGE.

#### 3.1.5. Correlation Analysis of PASC Patients of Statistically Different Parameters Between PC and HC Group

**Figure 1:**
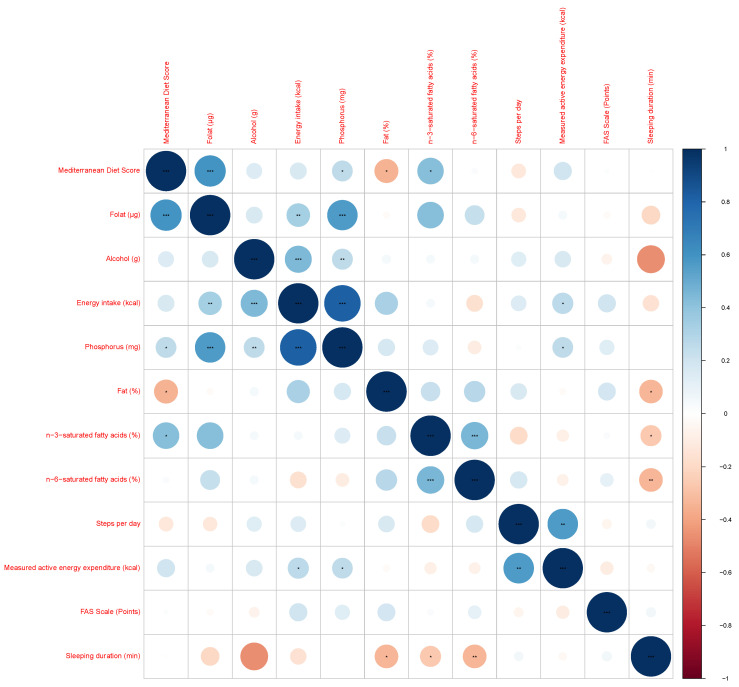
Correlation plot of PASC patients. Spearman´s coefficient has been used, and Bonferroni correction was applied for multiple comparisons. * *p* < 0.05, ** *p* < 0.01, *** *p* < 0.001.

Figure 1 shows the correlation matrix between the statistically significant different parameters between the PC and HC groups. Supplementary Table S1 provides details of the Spearman’s correlation coefficient. No significant correlation was found between the FAS and any of the other parameters. A negative correlation was found between sleep duration, the intake of fat, and saturated fatty acids. A positive correlation was found between phosphorus, folate, energy intake, and measured active energy expenditure. There was also a positive correlation between the intake of *n*-3 and *n*-6-saturated fatty acids and a negative correlation between the MDS and fat intake.

### 3.2. Subgroup Analysis

#### 3.2.1. Subgroup Analysis of the PC Group Based on FAS

**Figure 2:**
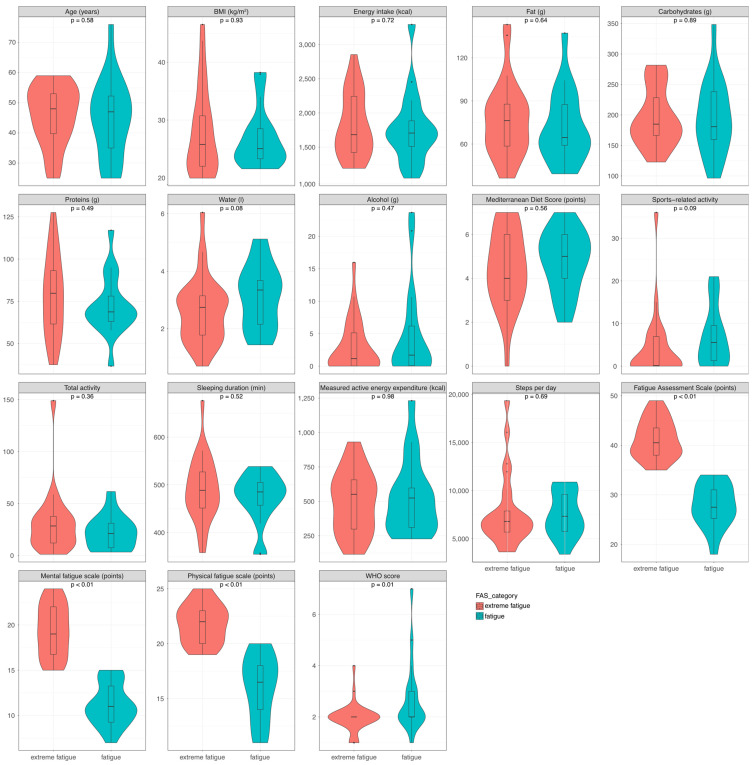
Facet violin box plots for subgroup analysis of PC patients. Facets show the different parameters. Red plots reflect patients with extreme fatigue (FAS ≥ 32 points), and green plots reflect patients with fatigue (FAS ≥ 22 and <35). Kruskal–Wallis Test was performed to test for significant differences between the groups. Abbreviation: BMI: body mass index; WHO: World Health Organisation.

Supplementary Table S2 shows details of the characteristics of the patients with fatigue divided into fatigue and extreme fatigue based on the score of the FAS (≥22 and <35 points: fatigue; ≥35 points: extreme fatigue). Figure 2 shows violin box plots of some key parameters, analogous to the comparison between the PC group and the HC group. There were no significant differences in sex, age, BMI, nutrition, or energy values between the groups. Furthermore, there was no difference in the prevalence of olfactory or gustatory impairment during the acute COVID-19, as 70% of patients with extreme fatigue and 72% of patients with fatigue suffered from this symptom (*p* = 0.89, for details, see Supplementary Table S2). Similarly, there were no differences between the groups in wearable device readings. The results of the Freiburger Activity Questionnaire did not differ between the groups. Not surprisingly, patients with extreme fatigue had significantly higher scores in both the mental and physical fatigue domains. Patients with fatigue revealed a statistically significant difference in the initial course of their COVID-19 compared to patients with extreme fatigue, as indicated by the World Health Organisation (WHO) severity score [29]. The score ranged from 1 (no limitations of activity) to 4 (oxygen by mask or nasal prongs) in patients with extreme fatigue and from 1 to 7 (mechanical ventilation and additional organ support) in patients with fatigue.

#### 3.2.2. Sex-Specific Subgroup Analysis

**Figure 3:**
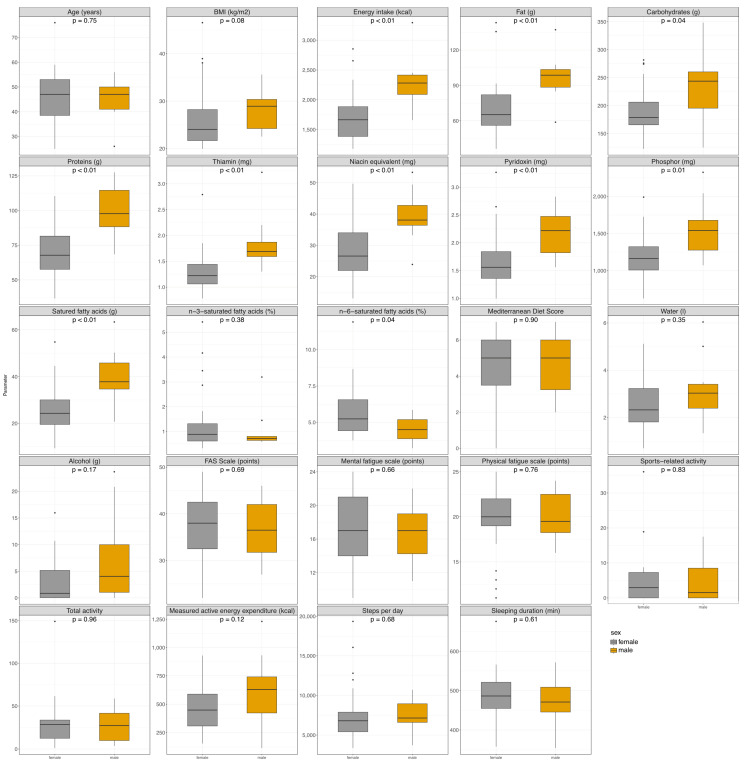
Facet box-plots of characteristics of sex-specific subgroup analysis of PC patients. Facet shows the different parameters: grey plots reflect male patients, and yellow plots reflect female patients. Kruskal–Wallis Test was performed to test for significant differences between the groups. Abbreviation: BMI: body mass index.

Sex-specific subgroup analysis was also performed for both PC and HC patients. In the PC group, there are 34 females and 12 males, and in the HC group, there are 35 females and 11 males were analyzed. Figure 3 shows the main parameters and significant findings in the PC group, and Supplementary Figure S1 shows these parameters for the HC group. Supplementary Tables S3 and S4 show all the details for these subgroup analyses.

In the PC group, male patients had a higher caloric intake, but there was no difference in the ratio. Male patients had a higher fat intake (g) (94 vs. 64g; *p* = 0.008), whereas female patients had a significantly higher intake of *n*-6-saturated fatty acids (4.5 vs. 5.2%; *p* = 0.036). There were no differences in the composition of saturated and unsaturated fat intake composition in the HC group. Male patients with fatigue had a higher protein intake in g but not in percentage. In addition, intakes of vitamins B1, B6, B12, niacin equivalents, sodium, phosphorus, and zinc intake were significantly higher in male patients (see Supplementary Table S3 for details). These findings were also observed in the HC group, so they may not be specific to post-COVID patients, but further differences in intake were observed in the HG group. Males also had a higher uptake of vitamin K, vitamin B2, potassium, calcium, magnesium, and copper, as well as cholesterol and alcohol. Healthy women had a higher percentage intake of sucrose. There was no difference detectable in activity or sleep duration in the PC group, but male participants in the HC group had a higher measured active energy expenditure.

## 4. Discussion

This study examined the daily food behavior, physical activity, and sleep duration of patients with PASC in comparison to an age- and sex-matched HC group.

The majority of patients with PASC who participated in this study were female; this is in line with the observation that women are at a higher risk of developing chronic fatigue syndrome [30].

No significant differences were observed in the overall caloric intake or the ratio of energy intake to the reference value. In light of these findings, it can be concluded that a reduction in caloric intake is unlikely to be a significant contributing factor to the observed increase in fatigue.

Fatigue is a common symptom of chronic inflammatory diseases such as sarcoidosis and rheumatic disorders [21,31]. It is noteworthy that PC patients had a higher dietary fat intake, especially of omega-3 and omega-6 fatty acids—polyunsaturated fatty acids (PUFA), but not of saturated fatty acids. PUFAs may play a key role in an anti-inflammatory diet. Several trials and a large narrative review have already addressed these research questions [32]. Omega-3 fatty acids are thought to have anti-inflammatory properties, whereas omega-6 fatty acids are thought to be pro-inflammatory [32,33]. Maes et al. demonstrated a negative correlation between a low omega-3:omega-6 ratio and fatigue severity in patients with chronic fatigue syndrome [34]. With regard to supplementation with specific polyunsaturated fatty acids, the study results of different studies are not consistent and do not all show a reduction in fatigue with omega-3 supplementation [32].

A systematic review has examined the effects of vitamins, magnesium, and trace elements on the various mechanisms contributing to the supportive treatment of PASC. In particular, PUFA, ascorbic acid, and vitamin D were identified as having significant potential for the treatment of symptoms and the prevention of severe disease [35]. The DGE recommends a maximum ratio of omega-6: omega-3 fatty acids of 5:1 [36]. Both groups did not quite reach this ratio, but again, the ratio was lower in the PC group, as a further sign of the healthier composition of the diet.

We also tested for a difference in adherence to the Mediterranean diet using the MDS. Although we did not find a significant difference, we observed a numerical trend with a higher score in PC patients (see Table 1, MDS median 5.0 in PC vs. 4.0 in HC).

Consolidating, we found a numerical trend in the implementation of the MDS in dietary behavior. There was also a trend, although not statistically significant, but also numerical, in the intake of dietary fiber. Both measures were higher in the PC group than in the HC group, indicating a possible trend toward healthier dietary habits in PASC patients. Since there is no causal therapy for the symptoms, only symptomatic relief, it is possible for patients to educate themselves about alternative options and leave no stone unturned to achieve improvement. Especially if it does not involve high costs and does not carry a high risk of unwanted side effects, It is possible that the group size was not large enough to reach statistical significance.

PC patients had a higher intake of vitamin C, well above the DGE recommendation. Previous studies have already shown a possible influence of vitamin C on the occurrence of fatigue and an improvement of fatigue symptoms with vitamin C [37,38,39]. The available evidence indicates that vitamin C supplementation exerts a beneficial effect on the symptoms, particularly on the perception of exertion in patients with PASC [40]. Supplementation is straightforward, and the products are readily available, thus encouraging PC patients with fatigue to take vitamin C supplements, with the expectation that they will be of benefit with minimal adverse effects. There was no difference in vitamin D intake between the PC and HC groups; both groups had intakes well below the recommendation levels. This is interesting in view of the worldwide vitamin D deficiency, especially in northern countries of the world [41]. Studies have not shown a beneficial effect in reducing respiratory infections in male patients but a worse outcome in patients with sepsis and severe deficiency compared with patients without vitamin D deficiency [42,43]. A large, non-randomized study found a high prevalence of low vitamin D in patients with fatigue. Symptoms improved significantly after serum vitamin D levels normalized [44]. The results of studies examining the relationship between vitamin D and PASC symptoms were found to be consistent [35,45].

In summary, an extensive review of the literature on diet and supplementation for the therapeutic management of chronic fatigue syndrome was unable to recommend a specific diet [46]. In addition, the NICE Guidelines for chronic fatigue syndrome focused more on advice from trained nutrition specialists and preventing malnutrition and weight loss [47,48].

Barrea and colleagues published a review of recommendations for post-COVID-19 syndrome; similarly, they did not recommend specific supplements but rather a balanced healthy diet, specifically a Mediterranean-style diet [49]. The diet comprises anti-inflammatory and antioxidant nutrients that exert immunomodulatory effects in diseases characterized by some degree of inflammatory activity [47,48,49]. Nevertheless, the current research regarding PASC indicates that determining micronutrient levels is a reasonable approach and that in cases of deficiency, supplementation may have an impact on PASC symptoms.

The SF-36 demonstrates a notable discrepancy between the PC and HC groups. Furthermore, this is significant when comparing extreme fatigue with fatigue in the PC group, with no gender difference. Fowler et al. demonstrate that fatigue, depressive symptoms, and a worsened health-related quality of life often co-occur in PSAC patients [50]. This lends support to the assertion that PASC patients are likely to be more inclined to implement changes due to the absence of evidence-based interventions. In particular, those that have minimal side effects and confer benefits, such as an approach to a healthier diet and micronutrient supplementation.

In terms of self-reported physical activity, both groups were statistically similar in how active they reported being in the past seven days. Based on the latest updated recommendations for adults from the American College of Sports Medicine and the American Heart Association, a minimum energy expenditure of 450 to 750 MET per week is considered necessary to have a positive impact on health [51]. When activities are converted to MET based on existing references, an assessment of exercise becomes possible [52]. Although there was no statistically significant difference between the groups, the clinical significance of the 10% difference in the number of patients receiving sufficient activity is noteworthy. In terms of measured steps per day, this is also not statistically different; however, patients with PASC took a median of 428 fewer steps per day (see Table 1). A large observational study in American women found decreasing all-cause mortality with an increasing number of steps per day, up to a limit of 7500 steps, beyond which mortality seemed to stabilize [53]. 1.79% of women with 6000–6999 steps per day died, whereas 1.16% of women with more than 7000 steps per day died during a follow-up of about 4 years, which is a relative risk reduction of at least 35% [53]. Another meta-analysis calculated a 7% reduction in cardiovascular disease for an increase of 500 steps per day and a 15% reduction in all-cause mortality for an increase of 1000 steps per day [54].

In addition to activity, sleep duration, and quality also affect cognitive and physical performance. Participants with PASC had a significantly longer sleep duration of 49 min per night. Patients with fatigue often report sleep disturbances and insomnia as one of the key symptoms of chronic fatigue syndrome [33]. It has been established that sleep disturbance occurs in patients with both mild, moderate, and severe symptoms [14]. Sleep disturbance has a relevant impact on quality of life and thus can also be a contributing factor to the diminished emotional well-being observed in the PC group [14,15]. Recently, Li and colleagues found the optimal sleep duration to be 7 h per night, so patients with fatigue may sleep too long, as this may also lead to cognitive impairment [55]. However, insomnia is also common in the general population, ranging from 6 to 31.2% in Europe [42]. One limitation is that we do not have detailed information on the duration of deep sleep as an important component of sleep quality. To date, there has been a paucity of large longitudinal studies investigating sleep behavior in PC patients using polygraphy or polysomnography.

Regarding the subgroup analysis of participants with fatigue and extreme fatigue, the sports-related activities were lower in the group with extreme fatigue, although there was no statistical significance, and no difference was observed in energy expenditure measured by the activity monitor. Total activity was higher in patients with extreme fatigue, although this was not statistically significant. Total activity levels were numerically higher in patients with extreme fatigue, although this was not statistically significant. There was no statistically significant difference in the Freiburg Questionnaire scores. Given the group division, it was expected that there would be a discrepancy between the patient groups in the assessment of the physical and mental fatigue subscales. However, the correlation analysis showed no statistically significant correlation between FAS and the different parameters tested.

Focusing on the gender subgroup analysis, several differences between women and men were found (see Supplementary Tables S3 and S4 for details). Differences in diet and eating habits between men and women have been previously published [56,57]. Overall, no sex-specific differences in dietary behavior were observed.

It should be noted that this study is limited by the lack of laboratory chemical analyses for vitamins and minerals. As a result, it is not possible to determine whether any of the subjects may have been deficient in these nutrients. In addition, we do not have FAS questionnaires from the HC group. In this regard, we cannot exclude the possibility that patients in the HC group also experienced fatigue. We do not have information on whether the patients followed a specific diet or on the overall quality of the diet. Because of the lack of causal treatment options for fatigue, patients may be receptive to alternative healing methods or dietary changes to feel like they can do something. As all questionnaires and the food diary are based on self-report, this has to be mentioned as a limitation as well. Moreover, we do not know if some of these patients may have been following specific behaviors, such as the Mediterranean diet. Finally, the data analysis was relatively exploratory. Without baseline values for both weight, physical activity, and dietary intake in the PC cohort prior to a COVID-19 infection, the data allow only limited evaluation. Possible reasons for the limited differences between the groups may be the selected observation period of seven days and the lack of adjustment for potential confounders in the selected analyses. The use of a historical pre-pandemic control group may also have confounded the analysis. Lastly, we did not perform an a priori sample size calculation due to the lack of appropriate studies in that field at the time.

## 5. Conclusions

Based on the results of this study, patients with fatigue related to PASC seem to have a qualitatively good diet, especially rich in unsaturated fatty acids. We could not identify any relevant malnutrition or reduced performance, neither on the basis of the patients’ own assessments using the Freiburger Activity Questionnaire nor on the basis of the wearable data. The only difference observed was in sleep duration, which may rather reflect one of the symptoms of fatigue. As these are rather preliminary findings, further research and larger longitudinal studies are warranted. One focus might be on sleep quality, which is warranted to assess whether improving sleep quality will also improve fatigue symptoms.

## Figures and Tables

**Table 1:** Summary of the statistical comparisons between PC and HC groups.

Method	Statistics	Sample (n)	Results
Group comparison of baseline characteristics, activity monitor data, Freiburger Activity Questionnaire, SF-36—emotional well-being	Pearson’s Chi-squared test; Kruskal-Wallis rank sum test	HC (46) vs. PC (46)	Table 2
Group comparison of macro- and micronutrients	Pearson’s Chi-squared test; Kruskal-Wallis rank sum test	HC (46) vs. PC (46)	Table 2
Correlation matrix of significant parameters of the group comparisons	Spearman’s correlation coefficient, Bonferroni adjustment	HC (46) vs. PC (46)	Figure 1
Subgroup comparison, shown as facet violin box-plots	Kruskal-Wallis test	Patients with Fatigue (18) and extreme fatigue (28) according to FAS	Figure 2
Subgroup comparison, shown as facet box-plots	Kruskal-Wallis test	Sex-specific subgroup of PC group (m = 12; w = 34)	Figure 3

## Data Availability

The original contributions presented in the study are included in the article, further inquiries can be directed to the corresponding author.

## Supplementary Materials

The following supporting information can be downloaded at: https://www.mdpi.com/article/10.3390/nu16234056/s1, Supplementary Table S1: Correlation matrix; Supplementary Table S2: Characteristics of PC group as a subgroup analysis of fatigue; Supplementary Table S3: Gender-specific characteristics of the HC group; Supplementary Table S4: Gender-specific characteristics of the HC group; Supplementary Figure S1: Facet violin plot for gender-specific subgroup analysis of HC group.
