# Supplementary material for: Exploring Physical Activity, Sleep, and Nutrition’s Role in Fatigue Among Post-COVID-19 Patients
Source: Nutrients. 2024 Nov 26;16(23):4056. doi: 10.3390/nu16234056 (PMC11643756; doi:10.3390/nu16234056)
Supplement: Supplementary file 1 [file nutrients-16-04056-s001.zip › nutrients-3324867-supplementary.pdf]

Article

Association between daily life activities, dietary intake and fatigue in post-COVID patients

Supplementary Material

Inhaltsverzeichnis

Supplementary Table 1. Correlation matrix 1

Supplementary Table 2. Characteristics of the PC group as subgroup analysis of fatigue 3

Supplementary Table 3. Gender-specific characteristics of the PC group 11

Supplementary Table 4. Gender-specific characteristics of the HC group 19

Supplementary Figure 1. Facet violin plot for gender-specific subgroup analysis of the HC group 26

Supplementary Table 1. Correlation matrix

Parameter	Phosphorus (mg)	Folat (µg)	Alcohol (g)	n-6-saturated fatty acids (%)	n-3-saturated fatty acids (%)	Fat (%)	Sleeping duration (min)	Measured active energy expenditure (kcal)	Steps per day	Energy intake (kcal)	Mediterranean Diet Score
FAS Scale (Points)	0.20098269	0.16836085	0.095066990	0.09774197	0.15254420	0.0907604	0.031684852	-0.00877194	-0.1220510	0.2619633	-0.008737055
Mediterranean Diet Score	0.25384034	0.56416833	0.124080049	0.02505644	0.40621537	-0.3368011	-0.007202245	0.19281440	-0.1247017	0.1844260	
Energy intake (kcal)	0.81288930	0.38415048	0.475335993	-0.18914585	0.09269195	0.2509405	-0.121111447	0.29326487	0.0742978		
Steps per day	-0.04938085	-0.20975536	0.066748264	0.17456962	-0.24041075	0.1265479	0.018121413	0.53412866			
Measured active energy expenditure (kcal)	0.26759287	0.06931441	0.207526421	-0.07897916	-0.07595892	-0.1090305	-0.072636666				

Parameter	Phosphorus (mg)	Folat (µg)	Alcohol (g)	n-6-saturated fatty acids (%)	n-3-saturated fatty acids (%)	Fat (%)	Sleeping duration (min)	Measured active energy expenditure (kcal)	Steps per day	Energy intake (kcal)	Mediterranean Diet Score
Sleeping duration (min)	0.03141045	-0.17381456	-0.390022243	-0.36937481	-0.23089701	-0.2848082					
Fat (%)	0.12377428	-0.04113475	-0.013556268	0.23885291	0.19333950						
n-3-saturated fatty acids (%)	0.22627197	0.52550108	0.082885128	0.41893309							
n-6-saturated fatty acids (%)	-0.16447734	0.17742831	0.005137764								
Alcohol (g)	0.27923436	0.20297262									
Folat (µg)	0.60690718										

Supplementary Table 2. Characteristics of the PC group as subgroup analysis of fatigue

	Extreme fatigue (N=28)	fatigue (N=18)	Total (N=46)	p value
**Sex**				0.31
Male	6 (21%)	6 (35%)	12 (27%)	
female	22 (79%)	11 (65%)	33 (73%)	
Missing	0	1	1	
**Age**				0.57[2]
Median (Q1, Q3)	48 (40, 53)	47 (33, 53)	47 (39, 53)	
Missing	0	1	1	
**Fatigue Assessment Scale [points]**				**< 0.001[2]**
Median (Q1, Q3)	41 (38, 44)	28 (25, 31)	38 (30, 42)	
Missing	0	0	0	
**Mental fatigue score [points]**				**< 0.001[2]**
Median (Q1, Q3)	19 (17, 22)	11 (9, 13)	16 (11, 20)	
Missing	0	0	0	
**Physical fatigue score [points]**				**< 0.001[2]**
Median (Q1, Q3)	22 (20, 23)	17 (14, 18)	20 (18, 22)	
Missing	0	0	0	
**WHO Score**				**0.01[2]**
Median (Q1, Q3)	2 (2, 2)	2 (2, 3)	2 (2, 2)	
Missing	1	0	1	
**Body mass index [kg/m²]**				0.89[2]
Median (Q1, Q3)	26 (22, 31)	25 (23, 29)	26 (23, 31)	
Missing	0	1	1	

**Reference value of energy intake [kcal]**				0.98²
Median (Q1, Q3)	2500 (2300, 2700)	2500 (2300, 2600)	2500 (2300, 2700)	
Missing	0	1	1	
**Energy intake [kcal]**				0.69²
Median (Q1, Q3)	1700 (1400, 2200)	1700 (1500, 1900)	1700 (1400, 2100)	
Missing	0	1	1	
**Energy intake / Reference value [Ratio]**				0.80²
Median (Q1, Q3)	0.7 (0.6, 0.8)	0.8 (0.6, 0.8)	0.7 (0.6, 0.8)	
Missing	0	1	1	
**Fat [%]**				0.71²
Median (Q1, Q3)	38 (32, 41)	36 (33, 40)	37 (33, 41)	
Missing	0	1	1	
**Fat [g]**				0.80²
Median (Q1, Q3)	76 (59, 88)	65 (59, 88)	68 (59, 88)	
Missing	0	1	1	
**Carbohydrates [%]**				0.66²
Median (Q1, Q3)	44 (40, 46)	45 (40, 48)	45 (40, 47)	
Missing	0	1	1	
**Carbohydrates [g]**				0.74²
Median (Q1, Q3)	190 (170, 230)	180 (160, 230)	180 (160, 230)	
Missing	0	1	1	
**Protein [%]**				0.85²
Median (Q1, Q3)	17 (15, 20)	17 (14, 18)	17 (15, 20)	
Missing	0	1	1	
**Protein [g]**				0.57²

Median (Q1, Q3)	80 (62, 93)	69 (65, 80)	72 (63, 88)	
Missing	0	1	1	
**Water [liter]**				0.08²
Median (Q1, Q3)	2.7 (1.8, 3.1)	3.4 (2.1, 3.7)	3.0 (1.8, 3.4)	
Missing	0	1	1	
**Retinol equivalent [mg]**				0.44²
Median (Q1, Q3)	1.0 (0.7, 1.7)	1.1 (0.8, 1.1)	1.0 (0.7, 1.4)	
Missing	0	1	1	
**Vitamin A (Retinol) [mg]**				0.51²
Median (Q1, Q3)	0.3 (0.2, 0.4)	0.3 (0.3, 0.5)	0.3 (0.2, 0.4)	
Missing	0	1	1	
**Vitamin D (Calciferol) [µg]**				0.38²
Median (Q1, Q3)	2.7 (1.6, 5.6)	4.5 (2.7, 6.2)	3.8 (1.6, 5.7)	
Missing	0	1	1	
**Vitamin E (Tocopherol) [mg]**				0.22²
Median (Q1, Q3)	12 (11, 16)	11 (10, 15)	12 (10, 16)	
Missing	0	1	1	
**Vitamin K [µg]**				0.80²
Median (Q1, Q3)	250 (200, 380)	280 (170, 330)	260 (190, 340)	
Missing	0	1	1	
**Vitamin B1 (Thiamin) [mg]**				0.06²
Median (Q1, Q3)	1.4 (1.2, 1.8)	1.2 (1.0, 1.3)	1.3 (1.1, 1.7)	
Missing	0	1	1	
**Vitamin B2 (Riboflavin) [mg]**				0.41²

Median (Q1, Q3)	1.38 (1.17, 1.62)	1.44 (1.29, 1.64)	1.39 (1.22, 1.64)	
Missing	0	1	1	
**Niacin equivalent [mg]**				0.48²
Median (Q1, Q3)	32 (22, 37)	26 (24, 31)	28 (24, 36)	
Missing	0	1	1	
**Pantothenic acid [mg]**				0.69²
Median (Q1, Q3)	4.3 (3.8, 5.3)	5.0 (3.9, 5.6)	4.4 (3.9, 5.6)	
Missing	0	1	1	
**Vitamin B6 (Pyridoxin) [mg]**				0.50²
Median (Q1, Q3)	1.6 (1.3, 2.2)	1.6 (1.3, 1.8)	1.6 (1.3, 1.9)	
Missing	0	1	1	
**Biotin [µg]**				0.64²
Median (Q1, Q3)	47 (37, 57)	50 (39, 59)	48 (37, 58)	
Missing	0	1	1	
**Folate [µg]**				0.80²
Median (Q1, Q3)	230 (200, 320)	240 (200, 290)	230 (200, 310)	
Missing	0	1	1	
**Vitamin B12 (Cobalamin) [µg]**				0.75²
Median (Q1, Q3)	5.0 (3.7, 6.8)	4.6 (4.0, 5.2)	4.8 (3.8, 6.5)	
Missing	0	1	1	
**Vitamin C (Ascorbic acid) [mg]**				0.64²
Median (Q1, Q3)	150 (110, 220)	160 (110, 180)	150 (110, 210)	
Missing	0	1	1	
**Sodium [g]**				0.78²
Median (Q1, Q3)	2.5 (2.0, 3.1)	2.6 (1.5, 3.4)	2.5 (1.9, 3.2)	
Missing	0	1	1	

**Potassium [g]**				0.98[2]
Median (Q1, Q3)	2.8 (2.4, 3.6)	3.1 (2.5, 3.2)	2.9 (2.4, 3.3)	
Missing	0	1	1	
**Calcium [g]**				0.29[2]
Median (Q1, Q3)	0.9 (0.7, 1.0)	1.0 (0.8, 1.2)	0.9 (0.7, 1.0)	
Missing	0	1	1	
**Magnesium [g]**				0.94[2]
Median (Q1, Q3)	0.3 (0.3, 0.4)	0.3 (0.3, 0.4)	0.3 (0.3, 0.4)	
Missing	0	1	1	
**Phosphorus [mg]**				0.78[2]
Median (Q1, Q3)	1200 (1000, 1400)	1200 (1100, 1400)	1200 (1000, 1400)	
Missing	0	1	1	
**Iron [mg]**				0.25[2]
Median (Q1, Q3)	12 (11, 15)	11 (10, 13)	12 (11, 14)	
Missing	0	1	1	
**Zinc [mg]**				0.25[2]
Median (Q1, Q3)	11 (9, 12)	10 (8, 11)	10 (8, 12)	
Missing	0	1	1	
**Copper [mg]**				0.80[2]
Median (Q1, Q3)	1.6 (1.5, 2.4)	1.8 (1.5, 2.2)	1.7 (1.5, 2.3)	
Missing	0	1	1	
**Manganese [mg]**				0.57[2]
Median (Q1, Q3)	4.2 (3.6, 6.8)	3.6 (3.5, 5.1)	4.2 (3.5, 5.6)	
Missing	0	1	1	
**Fluoride [mg]**				0.51[2]
Median (Q1, Q3)	0.8 (0.7, 1.1)	0.9 (0.7, 1.1)	0.9 (0.7, 1.1)	
Missing	0	1	1	
**Jod [µg]**				0.43[2]
Median (Q1, Q3)	86 (68, 158)	146 (78, 176)	89 (68, 166)	

Missing	0	1	1	
**Fiber [g]**				0.37²
Median (Q1, Q3)	22 (19, 31)	22 (18, 26)	22 (19, 28)	
Missing	0	1	1	
**Sucrose [%]**				0.15²
Median (Q1, Q3)	9 (7, 11)	12 (9, 13)	10 (8, 12)	
Missing	0	1	1	
**Sucrose [g]**				0.37²
Median (Q1, Q3)	42 (33, 52)	46 (38, 60)	434 (33, 53)	
Missing	0	1	1	
**Cholesterol [g]**				0.85²
Median (Q1, Q3)	0.3 (0.2, 0.4)	0.3 (0.2, 0.4)	0.3 (0.2, 0.4)	
Missing	0	1	1	
**Alcohol [g]**				0.54²
Median (Q1, Q3)	1.2 (0.0, 5.1)	1.7 (0.1, 6.6)	1.5 (0.0, 5.3)	
Missing	0	1	1	
**Saturated fatty acids [%]**				0.39²
Median (Q1, Q3)	14 (11, 15)	14 (12, 17)	14 (12, 16)	
Missing	0	1	1	
**Fatty acids [g]**				0.87²
Median (Q1, Q3)	27 (21, 33)	26 (21, 35)	27 (21, 35)	
Missing	0	1	1	
**N-3 Fatty acids (omega-3) [%]**				0.40²
Median (Q1, Q3)	0.9 (0.6, 1.2)	0.7 (0.6, 0.9)	0.8 (0.6, 1.1)	
Missing	0	1	1	
**N-6 Fatty acids (omega-6) [%]**				0.25²
Median (Q1, Q3)	5.2 (4.4, 6.5)	5.1 (4.0, 5.6)	5.1 (4.2, 6.1)	

Missing	0	1	1	
**Mediterranean Diet Score (points)**				0.78²
Median (Q1, Q3)	4 (3, 6)	5 (4, 6)	5 (3, 6)	
Missing	1	5	6	
**Freiburger-Questionnaire [hours/week]**				
**Sports-related activities**				0.10²
Median (Q1, Q3)	0 (0, 6.9)	4 (1, 10)	2 (0, 8)	
Missing	6	3	9	
**Total activity**				0.20²
Median (Q1, Q3)	28 (12, 38)	19 (6, 30)	23 (11, 33)	
Missing	7	4	11	
**Wearable data**				
**Measured active energy expenditure [kcal]**				0.87²
Median (Q1, Q3)	550 (300, 660)	540 (310, 600)	540 (310, 640)	
Missing	3	1	4	
**Steps per day [steps]**				0.64²
Median (Q1, Q3)	6800 (5700, 7900)	7500 (5600, 9600)	6700 (5700, 9500)	
Missing	3	1	4	
**Measured sleep duration [min]**				0.48²

Median (Q1, Q3)	490 (450, 530)	480 (460, 510)	490 (450, 520)
Missing	3	1	4

1. Pearson's Chi-squared test
2. Kruskal-Wallis rank sum test

Abbreviation: WHO: World Health Organisation Score

Supplementary Table 3. Gender-specific characteristics of the PC group

	Male (N=12)	Female (N=34)	Total (N=46)	p value
**Age [years]**				0.80[2]
Median (Q1, Q3)	47 (41, 51)	47 (38, 53)	47 (39, 53)	
Missing	0	0	0	
**Fatigue category**				0.37[2]
Extreme fatigue	6 (50%)	22 (65%)	28 (61%)	
fatigue	6 (50%)	12 (36%)	18 (39%)	
Missing	0	0	0	
**Fatigue Assessment Scale [points]**				0.35[2]
Median (Q1, Q3)	35 (29, 40)	38 (31, 42)	38 (30, 42)	
Missing	0	0	0	
**Mental Fatigue Score [points]**				0.37[2]
Median (Q1, Q3)	16 (11, 19)	16 (12, 21)	16 (11, 20)	
Missing	0	0	0	
**Physical Fatigue Score [points]**				0.37[2]
Median (Q1, Q3)	19 (16, 22)	20 (18, 22)	20 (18, 22)	
Missing	0	0	0	
**WHO Scale [points]**				0.50[2]
Median (Q1, Q3)	2 (2, 2)	2 (2, 2)	2 (2, 2)	
Missing	0	1	1	

**Body mass index [kg/m²]**				0.06[2]
Median (Q1, Q3)	29 (25, 32)	24 (22, 30)	25 (23, 30)	
Missing	0	0	0	
**Reference value of energy intake [kcal]**				**< 0.001[2]**
Median (Q1, Q3)	3200 (2700, 3500)	2400 (2300, 2500)	2500 (2300, 2700)	
Missing	0	0	0	
Energy **intake [kcal]**				**0.01[2]**
Median (Q1, Q3)	2200 (1800, 2400)	1600 (1400, 1900)	1700 (1400, 2100)	
Missing	0	0	0	
**Energy intake / Reference value [Ratio]**				0.92[2]
Median (Q1, Q3)	0.7 (0.5, 0.8)	0.7 (0.6, 0.8)	0.7 (0.6, 0.8)	
Missing	0	0	0	
**Fat [%]**				0.64[2]
Median (Q1, Q3)	37 (35, 40)	37 (32, 41)	37 (33, 41)	
Missing	0	0	0	
**Fat [g]**				**0.01[2]**
Median (Q1, Q3)	94 (79, 102)	64 (57, 81)	67 (59, 88)	
Missing	0	0	0	
**Carbohydrates [%]**				0.19[2]
Median (Q1, Q3)	43 (40, 45)	45 (40, 48)	45 (40, 48)	
Missing	0	0	0	
**Carbohydrates [g]**				0.07[2]

Median (Q1, Q3)	227.90 (183.16, 260.07)	177.89 (158.49, 204.61)	183.33 (164.76, 235.86)	
Missing	0	0	0	
**Protein [%]**				0.21[2]
Median (Q1, Q3)	17 (16, 21)	17 (14, 18)	17 (15, 20)	
Missing	0	0	0	
**Protein [g]**				**< 0.001[2]**
Median (Q1, Q3)	92 (82, 110)	68 (58, 81)	71 (63, 88)	
Missing	0	0	0	
**Water [liter]**				0.18[2]
Median (Q1, Q3)	3.1 (2.5, 3.5)	2.6 (1.8, 3.2)	3.0 (2.0, 3.4)	
Missing	0	0	0	
**Retinol equivalent [mg]**				1.00[2]
Median (Q1, Q3)	1.1 (0.6, 1.9)	1.0 (0.8, 1.2)	1.0 (0.7, 1.4)	
Missing	0	0	0	
**Vitamin A (Retinol) [mg]**				0.17[2]
Median (Q1, Q3)	0.4 (0.3, 0.5)	0.3 (0.2, 0.4)	0.3 (0.2, 0.4)	
Missing	0	0	0	
**Vitamin D (Calciferol) [µg]**				0.27[2]
Median (Q1, Q3)	5.6 (2.4, 6.9)	2.8 (1.6, 5.3)	3.7 (1.6, 5.7)	
Missing	0	0	0	
**Vitamin E (Tocopherol) [mg]**				0.14[2]
Median (Q1, Q3)	11 (9, 13)	12 (11, 17)	12 (10, 16)	

Missing	0	0	0	
**Vitamin K [µg]**				0.78[2]
Median (Q1, Q3)	250.05 (164.15, 333.93)	264.05 (195.30, 335.90)	263.25 (189.82, 335.90)	
Missing	0	0	0	
**Vitamin B1 (Thiamin) [mg]**				**0.01**[2]
Median (Q1, Q3)	1.6 (1.4, 1.9)	1.2 (1.1, 1.4)	1.3 (1.1, 1.6)	
Missing	0	0	0	
**Vitamin B2 (Riboflavin) [mg]**				0.06[2]
Median (Q1, Q3)	1.5 (1.3, 2.2)	1.4 (1.1, 1.6)	1.4 (1.2, 1.6)	
Missing	0	0	0	
**Niacin equivalent [mg]**				**0.002**[2]
Median (Q1, Q3)	38 (31, 42)	26 (22, 33)	28 (24, 36)	
Missing	0	0	0	
**Pantothenic acid [mg]**				0.14[2]
Median (Q1, Q3)	5.4 (3.9, 6.2)	4.2 (3.9, 5.0)	4.4 (3.9, 5.6)	
Missing	0	0	0	
**Vitamin B6 (Pyridoxin) [mg]**				**0.02**[2]
Median (Q1, Q3)	2.0 (1.6, 2.5)	1.5 (1.3, 1.8)	1.6 (1.3, 1.9)	
Missing	0	0	0	
**Biotin [µg]**				0.41[2]
Median (Q1, Q3)	49 (42, 66)	47 (37, 56)	49 (38, 58)	
Missing	0	0	0	

**Folate [µg]**				0.67[2]
Median (Q1, Q3)	260 (210, 329)	230 (200, 310)	230 (200, 310)	
Missing	0	0	0	
**Vitamin B12 (Cobalamin) [µg]**				**0.02[2]**
Median (Q1, Q3)	6.7 (4.2, 8.0)	4.4 (3.4, 5.2)	4.7 (3.8, 6.5)	
Missing	0	0	0	
**Vitamin C (Ascorbic acid) [mg]**				0.75[2]
Median (Q1, Q3)	150 (100, 200)	150 (110, 210)	150 (110, 210)	
Missing	0	0	0	
**Sodium [g]**				**0.01[2]**
Median (Q1, Q3)	3.2 (2.8, 3.5)	2.2 (1.5, 3.0)	2.5 (1.9, 3.2)	
Missing	0	0	0	
**Potassium [g]**				0.15[2]
Median (Q1, Q3)	3.4 (2.5, 4.1)	2.8 (2.4, 3.2)	2.9 (2.4, 3.4)	
Missing	0	0	0	
**Calcium [g]**				0.06[2]
Median (Q1, Q3)	1.0 (0.8, 1.3)	0.9 (0.7, 1.0)	0.9 (0.7, 1.0)	
Missing	0	0	0	
**Magnesium [g]**				0.09[2]
Median (Q1, Q3)	0.4 (0.4, 0.5)	0.3 (0.3, 0.4)	0.3 (0.3, 0.4)	
Missing	0	0	0	
**Phosphorus [mg]**				**0.02[2]**

Median (Q1, Q3)	1400 (1200, 1600)	1200 (1000, 1300)	1200 (1000, 1400)	
Missing	0	0	0	
**Iron [mg]**				0.11[2]
Median (Q1, Q3)	14 (11, 16)	12 (10, 13)	12 (11, 14)	
Missing	0	0	0	
**Zinc [mg]**				**0.002**[2]
Median (Q1, Q3)	13 (11, 15)	9 (8, 11)	10 (9, 12)	
Missing	0	0	0	
**Copper [mg]**				0.26[2]
Median (Q1, Q3)	2.1 (1.6, 2.5)	1.7 (1.5, 2.3)	1.8 (1.5, 2.4)	
Missing	0	0	0	
**Manganese [mg]**				0.27[2]
Median (Q1, Q3)	4.9 (3.6, 6.8)	4.1 (3.4, 5.4)	4.2 (3.5, 6.5)	
Missing	0	0	0	
**Fluoride [mg]**				0.32[2]
Median (Q1, Q3)	1.0 (0.8, 1.0)	0.8 (0.6, 1.2)	0.9 (0.7, 1.2)	
Missing	0	0	0	
**Jod [µg]**				0.86[2]
Median (Q1, Q3)	85 (74, 167)	90 (69, 165)	88 (69, 165)	
Missing	0	0	0	
**Fiber [g]**				0.53[2]
Median (Q1, Q3)	23 (19, 29)	22 (19, 28)	22 (19, 29)	

Missing	0	0	0	
**Sucrose [%]**				0.07[2]
Median (Q1, Q3)	9 (6, 11)	10 (9, 13)	10 (8, 12)	
Missing	0	0	0	
**Sucrose [g]**				0.92[2]
Median (Q1, Q3)	43 (32, 57)	43 (33, 53)	43 (33, 53)	
Missing	0	0	0	
**Cholesterol [g]**				0.08[2]
Median (Q1, Q3)	0.3 (0.2, 0.4)	0.3 (0.2, 0.3)	0.3 (0.2, 0.4)	
Missing	0	0	0	
**Alcohol [g]**				0.30[2]
Median (Q1, Q3)	2.9 (0.4, 8.6)	0.8 (0.0, 4.8)	1.6 (0.0, 5.2)	
Missing	0	0	0	
**Saturated fatty acids [%]**				0.15[2]
Median (Q1, Q3)	15 (14, 17)	13 (11, 16)	14 (11, 16)	
Missing	0	0	0	
**Saturated fatty acids [g]**				0.01[2]
Median (Q1, Q3)	36 (30, 43)	24 (20, 30)	27 (21, 35)	
Missing	0	0	0	
**N-3 Fatty acids (omega-3) [%]**				0.12[2]
Median (Q1, Q3)	0.7 (0.6, 0.8)	0.9 (0.6, 1.2)	0.8 (0.6, 1.1)	
Missing	0	0	0	

**N-6 Fatty acids (omega-6) [%]**				**0.04**[2]
Median (Q1, Q3)	4.5 (3.8, 5.3)	5.2 (4.4, 6.6)	5.2 (4.2, 6.2)	
Missing	0	0	0	
**Mediterranean Diet Score (points)**				0.90[2]
Median (Q1, Q3)	5 (3, 6)	5 (4, 6)	5 (3, 6)	
Missing	2	3	5	
**Freiburger-Questionnaire [hours/week]**				
**Sports-related activities**				0.93[2]
Median (Q1, Q3)	2 (0, 12)	3 (0, 7)	2 (0, 7)	
Missing	1	7	8	
Total activity				0.89[2]
Median (Q1, Q3)	27 (8, 36)	22 (12, 33)	25 (12, 34)	
Missing	2	8	10	
**Wearable data**				
**Measured active energy expenditure [kcal]**				0.09[2]
Median (Q1, Q3)	590 (500, 720)	410 (300, 590)	540 (310, 630)	
Missing	0	3	3	
**Steps per day [steps]**				0.37[2]
Median (Q1, Q3)	7600 (6600, 9600)	6800 (5500, 7800)	6800 (5700, 9500	
Missing	0	3	3	
**Measured sleep duration [min]**				0.48[2]
Median (Q1, Q3)	470 (440, 510)	490 (460, 520)	490 (450, 520)	

Missing	0	3	3	

1. Pearson's Chi-squared test
2. Kruskal-Wallis rank sum test

Abbreviation: WHO: World Health Organisation Score

Supplementary Table 4. Gender-specific characteristics of the HC group

	Male (N=11)	Female (N=35)	Total (N=46)	p value
**Age [years]**				0.99[2]
Median (Q1, Q3)	48 (41, 51)	47 (40, 54)	47 (40, 53)	
Missing	0	0	0	
**Body mass index [kg/m²]**				0.13[2]
Median (Q1, Q3)	28 (26, 30)	24 (22, 29)	26 (23, 30)	
Missing	0	0	0	
**Reference value of energy intake [kcal]**				**< 0.001[2]**
Median (Q1, Q3)	3000 (2800, 3200)	2300 (2100, 2500)	2400 (2100, 2700)	
Missing	0	1	1	
**Energy intake [kcal]**				**0.01[2]**
Median (Q1, Q3)	2100 (1700, 2500)	1600 (1300, 1900)	1800 (1400, 2100)	
Missing	0	1	1	
**Energy intake / Reference value [Ratio]**				0.94[2]
Median (Q1, Q3)	0.7 (0.6, 0.8)	0.7 (0.6, 0.9)	0.7 (0.6, 0.9)	
Missing	0	1	1	

**Fat [%]**				0.53[2]
Median (Q1, Q3)	35 (32, 37)	33 (30, 37)	34 (30, 37)	
Missing	0	1	1	
**Fat [g]**				**0.02[2]**
Median (Q1, Q3)	72 (61, 100)	61 (49, 72)	64 (51, 75)	
Missing	0	1	1	
**Carbohydrates [%]**				0.05[2]
Median (Q1, Q3)	42 (37, 45)	47 (42, 49)	46 (41, 49)	
Missing	0	1	1	
**Carbohydrates [g]**				0.10[2]
Median (Q1, Q3)	200 (180, 250)	170 (150, 230)	180 (150, 240)	
Missing	0	1	1	
**Protein [%]**				0.05[2]
Median (Q1, Q3)	19 (17, 21)	18 (15, 18)	18 (16, 19)	
Missing	0	1	1	
**Protein [g]**				**< 0.001[2]**
Median (Q1, Q3)	98 (87, 110)	69 (58, 79)	72 (60, 89)	
Missing	0	1	1	
**Water [liter]**				0.32[2]
Median (Q1, Q3)	3.0 (2.1, 4.7)	2.8 (2.2, 3.2)	2.8 (2.2, 3.3)	
Missing	0	1	1	
**Retinol equivalent [mg]**				0.34[2]
Median (Q1, Q3)	1.1 (0.8, 1.7)	1.0 (0.7, 1.2)	1.0 (0.7, 1.3)	
Missing	0	1	1	
**Vitamin A (Retinol) [mg]**				0.67[2]
Median (Q1, Q3)	0.3 (0.2, 0.4)	0.3 (0.2, 0.4)	0.3 (0.2, 0.4)	
Missing	0	1	1	
**Vitamin D (Calciferol) [µg]**				0.09[2]

Median (Q1, Q3)	5.9 (2.3, 7.6)	2.5 (1.5, 4.0)	2.5 (1.6, 5.8)	
Missing	0	1	1	
**Vitamin E (Tocopherol) [mg]**				0.24[2]
Median (Q1, Q3)	13 (9, 19)	11 (9, 13)	11 (9, 14)	
Missing	0	1	1	
**Vitamin K [µg]**				**0.00[2]**
Median (Q1, Q3)	420 (270, 580)	170 (150, 260)	220 (160, 310)	
Missing	0	1	1	
**Vitamin B1 (Thiamin) [mg]**				**0.03[2]**
Median (Q1, Q3)	1.5 (1.3, 1.9)	1.2 (0.9, 1.5)	1.2 (1.0, 1.6)	
Missing	0	1	1	
**Vitamin B2 (Riboflavin) [mg]**				**0.01[2]**
Median (Q1, Q3)	1.7 (1.5, 2.3)	1.4 (1.1, 1.6)	1.4 (1.3, 1.7)	
Missing	0	1	1	
**Niacin equivalent [mg]**				**< 0.001[2]**
Median (Q1, Q3)	39 (34, 44)	27 (23, 30)	28 (25, 33)	
Missing	0	1	1	
**Pantothenic acid [mg]**				**0.00[2]**
Median (Q1, Q3)	5.5 (4.7, 6.2)	4.0 (3.5, 4.6)	4.3 (3.7, 5.3)	
Missing	0	1	1	
**Vitamin B6 (Pyridoxin) [mg]**				**< 0.001[2]**
Median (Q1, Q3)	2.0 (1.7, 2.4)	1.4 (1.2, 1.6)	1.5 (1.3, 1.7)	
Missing	0	1	1	
**Biotin [µg]**				0.10[2]
Median (Q1, Q3)	50 (41, 59)	43 (36, 52)	44 (35, 55)	

Missing	0	1	1	
**Folate [µg]**				0.28²
Median (Q1, Q3)	193.70 (0.42, 425.55)	180.70 (0.27, 236.75)	188.20 (0.27, 239.60)	
Missing	0	1	1	
**Vitamin B12 (Cobalamin) [µg]**				**0.00²**
Median (Q1, Q3)	7 (6, 10)	4 (3, 6)	5 (3, 6)	
Missing	0	1	1	
**Vitamin C (Ascorbic acid) [mg]**				0.17²
Median (Q1, Q3)	153 (108, 172)	116 (87, 160	116 (89, 163)	
Missing	0	1	1	
**Sodium [g]**				**0.02²**
Median (Q1, Q3)	3.0 (2.8, 3.6)	2.4 (2.1, 2.9)	2.7 (2.3, 3.0)	
Missing	0	1	1	
**Potassium [g]**				**0.00²**
Median (Q1, Q3)	3.5 (2.9, 4.3)	2.6 (2.3, 3.0)	2.7 (2.4, 3.1)	
Missing	0	1	1	
**Calcium [g]**				**0.06²**
Median (Q1, Q3)	1.1 (0.9, 1.4)	0.8 (0.7, 1.1)	0.9 (0.8, 1.1)	
Missing	0	1	1	
**Magnesium [g]**				**0.01²**
Median (Q1, Q3)	0.4 (0.4, 0.5)	0.3 (0.3, 0.4)	0.3 (0.3, 0.4)	
Missing	0	1	1	
**Phosphorus [mg]**				0.14²
Median (Q1, Q3)	1300 (2, 1700)	1100 (1, 1300)	1100 (2, 1300)	
Missing	0	1	1	
**Iron [mg]**				0.06²
Median (Q1, Q3)	15 (10, 19)	11 (9, 12)	12 (10, 14)	
Missing	0	1	1	

**Zinc [mg]**				**0.00**[2]
Median (Q1, Q3)	13.08 (10.80, 17.28)	9.83 (7.89, 11.39)	10.50 (8.14, 12.01)	
Missing	0	1	1	
**Copper [mg]**				**0.01**[2]
Median (Q1, Q3)	2.3 (1.8, 3.1)	1.7 (1.5, 1.9)	1.8 (1.6, 2.1)	
Missing	0	1	1	
**Manganese [mg]**				0.37[2]
Median (Q1, Q3)	4.4 (3.7, 10.8)	4.2 (3.6, 5.6)	4.3 (3.6, 5.6)	
Missing	0	1	1	
**Fluoride [mg]**				0.56[2]
Median (Q1, Q3)	1.0 (0.7, 1.3)	0.9 (0.8, 1.1)	0.9 (0.7, 1.1)	
Missing	0	1	1	
**Jod [µg]**				1.00[2]
Median (Q1, Q3)	112 (0, 117)	102 (0, 142)	109 (0, 142)	
Missing	0	1	1	
**Fiber [g]**				0.37[2]
Median (Q1, Q3)	21 (17, 31)	20 (17, 23)	20 (17, 24)	
Missing	0	1	1	
**Sucrose [%]**				**0.00**[2]
Median (Q1, Q3)	7 (6, 10)	11 (8, 13)	10 (7, 13)	
Missing	0	1	1	
**Sucrose [g]**				0.43[2]
Median (Q1, Q3)	40 (26, 44)	41 (33, 59)	40 (28, 57)	
Missing	0	1	1	
**Cholesterol [g]**				**0.03**[2]
Median (Q1, Q3)	0.4 (0.2, 0.4)	0.3 (0.2, 0.3)	0.3 (0.2, 0.4)	
Missing	0	1	1	
**Alcohol [g]**				**0.00**[2]
Median (Q1, Q3)	8 (5, 14)	2 (0, 6)	3 (0, 8)	
Missing	0	1	1	
**Saturated fatty acids [%]**				0.79[2]

Median (Q1, Q3)	13.63 (12.26, 15.02)	13.53 (10.02, 15.47)	13.63 (11.58, 15.48)	
Missing	0	1	1	
**Saturated fatty acids [g]**				0.08²
Median (Q1, Q3)	29 (23, 43)	23 (19, 31)	26 (20, 32)	
Missing	0	1	1	
**N-3 Fatty acids (omega-3) [%]**				0.22²
Median (Q1, Q3)	0.8 (0.6, 0.9)	0.7 (0.5, 0.8)	0.7 (0.5, 0.8)	
Missing	0	1	1	
**N-6 Fatty acids (omega-6) [%]**				0.40²
Median (Q1, Q3)	4.3 (3.7, 4.5)	4.5 (3.8, 5.4)	4.5 (3.8, 5.2)	
Missing	0	1	1	
**Mediterranean Diet Score [points]**				0.27²
Median (Q1, Q3)	4 (3, 5)	4 (4, 5)	4 (3, 5)	
Missing	1	19	20	
**Freiburger Activity Questionnaire [hours/week]**				
**Sports-related activities**				0.08²
Median (Q1, Q3)	0 (0, 7)	5 (0, 19)	3 (0, 16)	
Missing	0	2	2	
**Total activity**				0.32²
Median (Q1, Q3)	22 (16, 27)	27 (16, 37)	26 (16, 36)	
Missing	0	2	2	

**Wearable data**				
**Measured active energy expenditure [kcal]**				**0.01**[2]
Median (Q1, Q3)	910 (600, 1100)	370 (300, 550)	430 (320, 750)	
Missing	3	12	15	
**Steps per day [steps]**				0.89[2]
Median (Q1, Q3)	7000 (6500, 9500)	7400 (6200, 11000)	7300 (6200, 11000)	
Missing	3	12	15	
**Measured sleep duration [min]**				0.71[2]
Median (Q1, Q3)	400 (350, 530)	430 (390, 470)	430 (390, 480)	
Missing	6	13	19	

1. Pearson's Chi-squared test
2. Kruskal-Wallis rank sum test

Abbreviation: WHO: World Health Organisation Score

Supplementary Figure 1. Facet violin plot for gender-specific subgroup analysis of the HC group

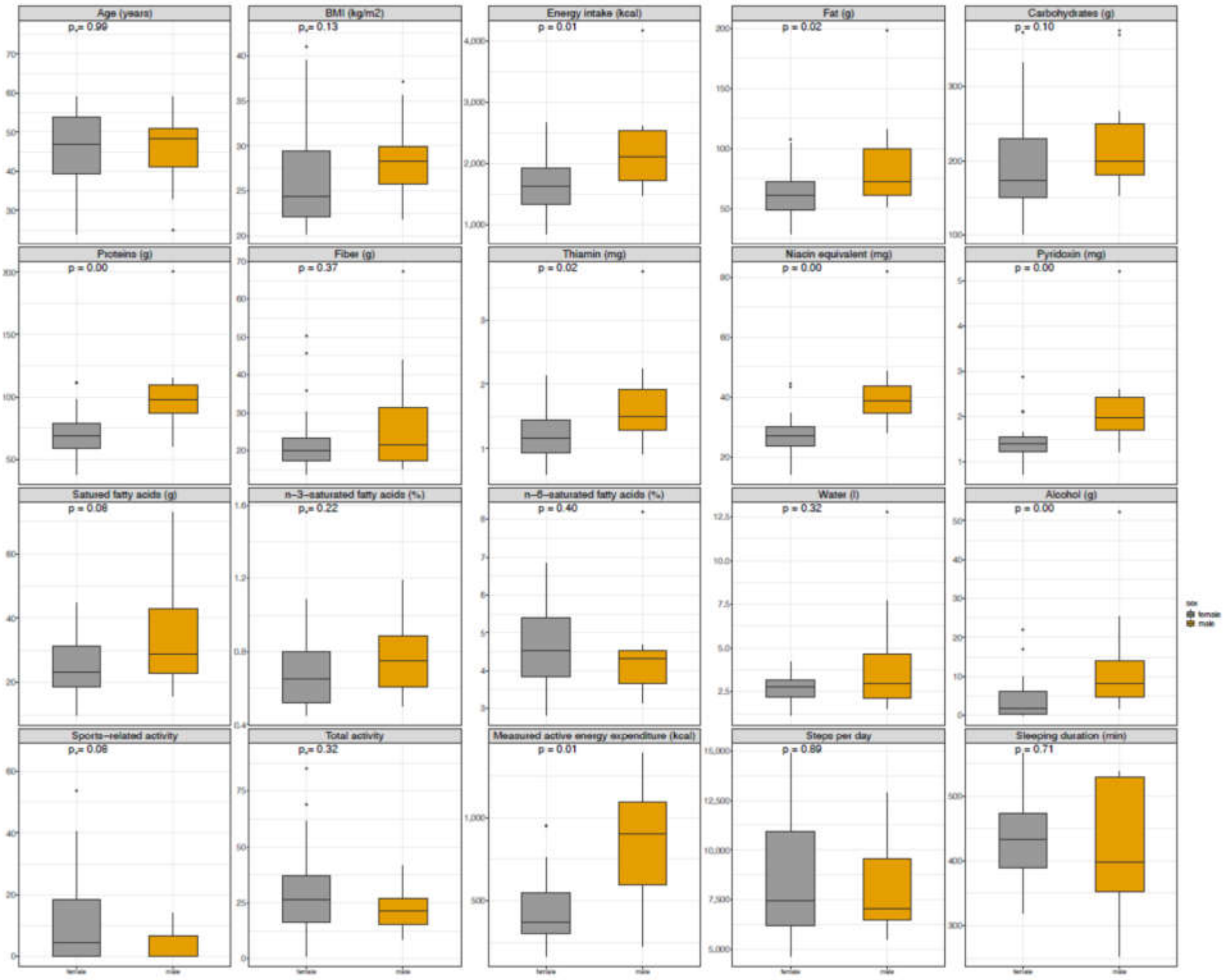

Facet violin box-plots of characteristics of sex-specific subgroup analysis of HC group. Facet show the different parameters, grey plots reflect male patients, yellow plots reflect female patients. Kruskal-Wallis-Test was performed to test for significant differences between the groups. *Abbreviation: BMI: body mass index; g: gram; l: liter; min: minutes; kcal: kilocalorie*
